# Detection of tmRNA molecules on microarrays at low temperatures using helper oligonucleotides

**DOI:** 10.1186/1472-6750-10-34

**Published:** 2010-04-28

**Authors:** Lauris Kaplinski, Ott Scheler, Sven Parkel, Priit Palta, Kadri Toome, Ants Kurg, Maido Remm

**Affiliations:** 1 Department of Bioinformatics, Institute of Molecular and Cell Biology, University of Tartu, Riia 23, Tartu, 51010, Estonia; 2 Estonian Biocentre, Riia 23b, Tartu, 51010, Estonia; 3 Department of Biotechnology, Institute of Molecular and Cell Biology, University of Tartu, Riia 23, Tartu, 51010, Estonia

## Abstract

**Background:**

The hybridization of synthetic *Streptococcus pneumoniae *tmRNA on a detection microarray is slow at 34°C resulting in low signal intensities.

**Results:**

We demonstrate that adding specific DNA helper oligonucleotides (chaperones) to the hybridization buffer increases the signal strength at a given temperature and thus makes the specific detection of *Streptococcus pneumoniae *tmRNA more sensitive. No loss of specificity was observed at low temperatures compared to hybridization at 46°C. The effect of the chaperones can be explained by disruption of the strong secondary and tertiary structure of the target RNA by the selective hybridization of helper molecules. The amplification of the hybridization signal strength by chaperones is not necessarily local; we observed increased signal intensities in both local and distant regions of the target molecule.

**Conclusions:**

The sensitivity of the detection of tmRNA at low temperature can be increased by chaperone oligonucleotides. Due to the complexity of RNA secondary and tertiary structures the effect of any individual chaperone is currently not predictable.

## Background

Over the last decade microarrays have quickly found applications in microbial diagnostics, for detecting different pathogenic viruses, bacteria and other microbes [1] or for analyzing species composition in environmental and medical samples [2]. Also, many different biosensor technologies based on nucleic acid hybridization have been developed and proposed for quick and cost effective "in-the-field" detection and identification of diseases, pathogens or contaminants [3,4].

The most common target molecule for diagnostic and phylogenetic studies is 16S rRNA (or corresponding gene). It was used in the 1970s [5] and continues to be the most widely-used marker for discriminating bacterial species [2,6]. The advantages of ribosomal small subunit RNA are its presence in all species in high copy numbers and the different evolutionary rates of different regions of 16S rRNA, making various taxonomic studies possible [7,8]. Nevertheless, alternative marker molecules [9-12] have to be considered in case 16S rRNA is not suitable for precise detection and distinguishing between closely related species [13].

One interesting novel marker that has shown great potential in molecular diagnostics is the tmRNA transcript of bacterial *ssrA gene*. In living cells, tmRNA is present in relatively high copy numbers (around 1000 molecules per cell) [14,15] and is responsible for assisting ribosomes during translation when protein synthesis stalls. tmRNA molecules contain regions of species-specific sequence heterogeneity and can therefore be successfully used as markers for bacterial diagnostics [16,17].

Nucleic acid hybridizations in microbiology and molecular diagnostics have been performed at various temperatures ranging from 4°C and RT to around 40°C or even higher (50°C and above). It is suggested in previous studies, that the hybridization of complex target molecules is hindered below 42°C, leading e.g. to low signal intensities and bad probe specificity [18]. Low temperature hybridization is of great interest for emerging technologies, such as membrane biosensors, where the denaturation of membranes and proteins have to be avoided and "laboratory-on-chip" and embedded solutions, where maintaining different compartments with varying temperature can be complicated and costly. Modern oligonucleotide design tools allow the hybridization affinity and specificity of local regions to be estimated quite precisely at different temperatures. One of our main goals was to develop a hybridization method that would be suitable for use below 37°C.

Several difficulties can arise in the detection of bacterial RNA by hybridization. Target RNA degradation has to be prevented and nonspecific hybridizations with wrong targets avoided. The latter is rather difficult on highly conserved RNA molecules [19]. Strong secondary structures can block the hybridization sites inside the molecule and thus prevent hybridization almost completely or retard it significantly [20,21]. The secondary structure of RNA is much stronger than that of the corresponding DNA [22] and the detection of RNA is more difficult [23]. It is suggested that secondary structure may be the main reason why hybridization-based detection fails at room temperature [18,21] and it has to be disrupted, or (in the case of synthetic molecules) its formation has to be minimized, to gain access to the target regions of the RNA molecule [21,24]. The latter is especially crucial in the case of rRNA and tmRNA molecules as they both fold into complex secondary and tertiary structures.

For certain applications it is also important to be able to estimate the relative or absolute abundances of different bacterial strains or species quantitatively. Quantification of hybridization poses additional challenges, especially if the process is too slow to reach equilibrium.

Several approaches to improve the efficiency of hybridization have been described. The hybridization temperature can be increased. It is predicted that while about 70% of a 70-mer cDNA molecule is inaccessible at 42°C, only 30% of DNA and 50% of RNA remains inaccessible at 65°C [20]. Some authors have suggested that a higher temperature increases hybridization specificity, but other authors have found no such effect [23]. Designing probes for specific exposed areas of the molecule also increases the hybridization efficiency [20]. Measuring or predicting the effect of secondary structure is difficult [25,26], especially as the parts of molecule that do not form double-helical stems can themselves be blocked by higher-level structures [20]. Cleaving target molecules to smaller fragments is one widely-used option; it can expose most hybridization sites that are normally blocked [20,25].

Alternatively, specific helper oligonucleotides (chaperones) can be added to the hybridization solution to increase hybridization efficiency [18,23,27]. These molecules bind to target molecules and block specific or nonspecific intramolecular interactions that cause secondary structure formation. Chaperones are specific to certain target molecules and they also increase the specificity of hybridization. Chaperones can also be marked with fluorophores or other detectable markers, solving the problem of detecting hybridized intact RNA [23,27]. It is reported that chaperones immediately side-to-side with hybridization probes are most effective in increasing the effectiveness of hybridization at low temperature [28]. This has been explained by the prevention of hairpin structure formation and by the effect of base stacking between capture probe and chaperone [27].

In this study we evaluated the effect of short DNA helper oligonucleotides (chaperones) on the hybridization of synthetic *Streptococcus pneumoniae *tmRNA molecules to DNA microarray probes. The practical objective was to find an improved protocol for detecting bacterial species by tmRNA hybridization. The theoretical objective was to elucidate the effect of the complex structure of a longer RNA target molecule on hybridization kinetics. In addition, a practical use for chaperones as a interesting novel tool in secondary structure analysis was demonstrated.

## Results

### Weak hybridization signals at low temperature

**Figure 1:**
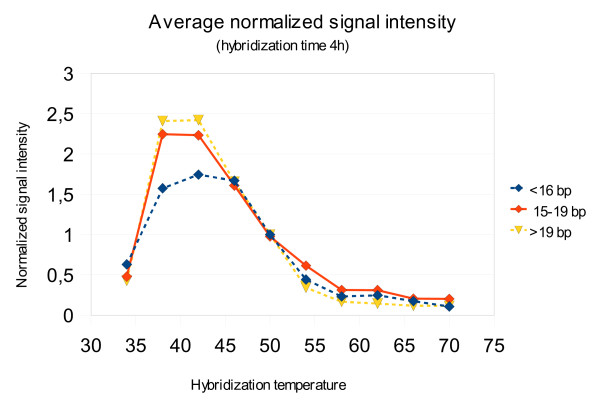
**The average signal intensities over the full range of hybridization temperatures**. The values are normalized by the signal intensity at 50°C to eliminate differences between the absolute signal values. <16 bp - the average signal intensity of probes shorter than 16 nucleotides. 15-19 bp - the average signal intensity of probes from 15 and 19 nucleotides long. >19 bp - the average signal intensity of probes at least 20 nucleotides long.

To determine the signal intensity and specificity over a range of temperatures, we performed hybridization experiments with synthetic *S. pneumoniae *tmRNA at temperatures ranging from 34°C to 72°C with 4°C steps. At temperatures below 42°C the relative signal intensities were lower than expected from the theoretical melting curves (Figure 1). On average, the decrease in relative signal intensity was more apparent for longer probes, but still clearly present even for probes only 9-10 nucleotides long.

The specificity of hybridization at 34°C was determined by analyzing signals from 21 probes on the same microarray, designed to the tmRNA sequences of other bacteria. We excluded 4 nonspecific probes, that had detectable false signals at 46°C, possibly due to errors in probe design process. The signal intensities of the remaining nonspecific probes were at least 200 times lower than the average intensity of the specific probes at and below 46°C.

To determine whether the weak signals at low temperatures were caused by slow hybridization or by a shift in equilibrium towards the secondary structure of the target molecules, a series of experiments were performed at 34°C by varying the hybridization time from 2 h to 12 h. The signal intensities increased with time but did not reach a plateau even after 12 h at 34°C. This indicated that hybridization did not reach equilibrium and the low signal intensities were probably caused by a slow rate.

### Chaperones increase the hybridization intensity at low temperatures

To determine whether the hybridization intensity at low temperatures can be increased by disrupting the tmRNA secondary structure, we added a mixture of six chaperone oligonucleotides in equal concentrations to the tmRNA solution before hybridization. Three different total chaperone concentrations were tested: 10, 100 and 1000 times the molar concentration of tmRNA. In all cases we recorded significant changes in the intensities of individual signals, which increased 2-3 times on average, although signals were strongly suppressed in several parts of the tmRNA molecule. We observed the highest increase of signal intensity with a relative concentration of chaperones to tmRNA = 100:1. At a relative concentration of 1000:1, the average signal strength was lower than at the 100:1 concentration. For all subsequent experiments we used a chaperone:tmRNA ratio of 100:1.

As expected, the signal intensities increased most markedly for probes that overlapped no chaperone. Most signals of overlapping probes were suppressed by chaperones, although few were higher.

To determine the effect of chaperones on probe specificity, we compared the signals of the *S. pneumoniae *probes with the signals of probes designed for other bacterial species. If the 4 probes, that had detectable false signals at 46°C, were removed, the difference between specific and nonspecific signals was more than 300-fold.

### Chaperones increase signal intensities in distantly located regions of the target molecule

**Figure 2:**
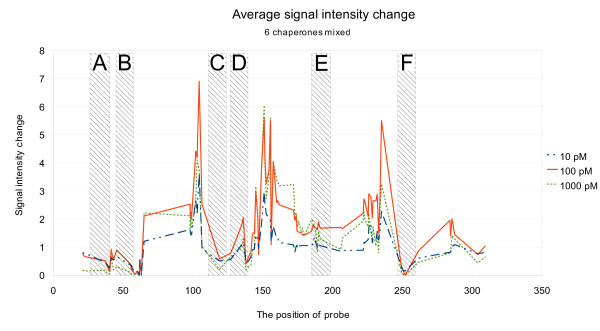
**The average relative signal intensity of all probes at different chaperone concentrations**. The signal intensities are arranged by the probe midpoint position on tmRNA. The hybridization regions of all chaperones are marked by shaded rectangles.

To determine which regions of tmRNA were affected by the presence of all six chaperones, we arranged the signals by the probe midpoint position on tmRNA. Three clearly outstanding regions of increased signal strength were apparent around nucleotide positions 100, 150 and 240 of tmRNA (Figure 2). These regions fall outside the chaperone hybridization areas on tmRNA.

**Figure 3:**
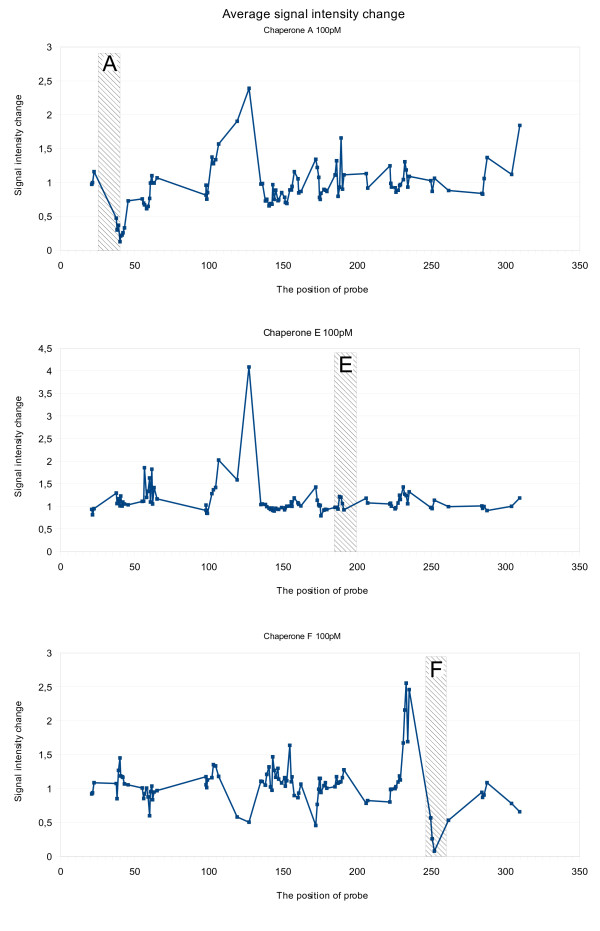
**The relative signal intensity change in the presence of different chaperones**. The signal intensities are arranged by the probe midpoint position on tmRNA. The shaded area is the hybridization region of the chaperone on tmRNA.

By hybridizing the tmRNA in the presence of individual chaperones and arranging the signals by probe midpoint position, we determined the regions most strongly affected by individual chaperones (Figure 3). Chaperone F amplified the signal intensities most strongly in the region (230-240) close to the chaperone hybridizing site (247-260), while chaperones A and E amplified the signals of distant regions. Chaperone A, binding to region 32-46, amplified signals 50 bp towards the 3' end of the tmRNA and also at the 3' end of the molecule. Chaperone E, binding to region 187-201, amplified signals 50 bp towards the 5' end. All chaperones strongly suppressed the signals of overlapped probes, except chaperone E.

We also calculated the probability of hybridization of each nucleotide in tmRNA using sFold and compared it with our hybridization diagrams. There was no significant correlation between the regions of greatly amplified signal strength and the regions of high probability of nucleotide pairing.

## Discussion

Ideally, hybridization can be viewed as an equilibrium between free and bound target molecules. The relative abundances of molecules in both states are determined by the difference between rates of duplex formation and dissociation. These rates in turn are determined by the free energy changes in the corresponding processes. Signal intensity is directly proportional to the number of molecules hybridized and is thus determined by the difference in free energy change between hybridization and dissociation. Equilibrium is usually preferred for hybridization because the signal strength is greatest, since the maximum numbers of target molecules are bound to the probes. Also, as hybridization rates may differ among molecules, only the equilibrium state guarantees that actual signal intensities correlate with the concentrations and hybridization affinities of the target molecules. If equilibrium is not reached, a rapidly but weakly hybridizing target may give a stronger signal than a slowly but strongly hybridizing one, so its relative concentration is overestimated.

At low temperature hybridization experiments, two important factors can influence the signal intensities. Both of them are strongly influenced by secondary structures of the target molecule. First, the equilibrium can change and the probe cannot compete with the secondary structure any more [21,26]. Second, the concentration of accessible target configurations is lower at low temperature due to the increased stability of tightly packed secondary structures. The hybridization rate is proportional to the concentration of accessible target molecules and is therefore much slower at low temperatures [26].

If the actual rate of hybridization is low, we may not reach equilibrium during the experiment. In that case the signals are initially low but increase over time. As we detected such behavior in our experiment, we concluded that the low signal intensities at low temperature are at least partially caused by slow hybridization.

By hybridizing with the target molecule, chaperones block the formation of at least some secondary or tertiary structure variants and thus increase the rate of hybridization at low temperatures [21,24]. Although it is shown, that designing chaperone specifically to the the region that forms intramolecular bonds increases hybridization signals significantly [28], this is not always possible.

We were unable to establish any correlation between the pairing probability of individual nucleotides in tmRNA, calculated with sFold, and the relative amplification of the signals in the presence of chaperones. This can be explained by the effect of tmRNA tertiary structure and the non-equilibrium state. The hybridization probability calculated by sFold only takes direct intramolecular pairings between nucleotides into account; it does not consider the blocking of potential hybridization sites by the globular structure of the molecule. Also, the probabilities of different conformations are calculated by absolute free energy levels, not taking into account the kinetics of secondary structure formation. Some conformations with low free energy may form very slowly, simply because they have to cross an unfavorable intermediate state.

When probe hybridization is blocked by the formation of a hairpin-like structure, the chaperone for the immediate neighborhood should work best because it does not allow the hairpin to form. It has been demonstrated that designing chaperones for the immediate neighborhood should work best if the accessibility of the region is hindered by the formation of secondary structure elements such as hairpins [28].

We were able to see such an effect with chaperone F, where the hybridization profiles of all probes suggest the presence of a hairpin. The chaperone binds to region 248-261 and the signals in the immediately preceding region (230-245) are strongly amplified in its presence. Thus, one can infer that without treatment, these regions are probably hybridized, forming a hairpin. Indeed, the ΔG plot generated by mFold suggests that there is a local energetic minimum between those regions. Nevertheless, it is important to notice that according to mFold this hairpin was not present in the most stable molecule conformation, as determined by the global energy minimum. Also, many other places with similar local energy profiles did not show a similar hybridization pattern. Thus, a hairpin, even if energetically favorable, may often not be the prevailing secondary structure pattern.

A high-level (tertiary) structure of RNA may also form by intramolecular hybridization between distant parts of a longer sequence. Thus the sites that affect the accessibility of a certain part of the molecule may be spatially separated from it. In that case, designing a chaperone for the immediate proximity of the probe may not work, because it either cannot hybridize because of blockage by the three-dimensional structure, or fails to make the neighboring site accessible because it is blocked by some other region. In that case, the best results should be obtained by a chaperone hybridizing specifically to the tertiary structure-forming region, so the tertiary structure cannot form and the conformation of the molecule is loosened. Such chaperones can potentially increase the probe hybridization rate in many regions of the molecule.

In the current experiments, the effect of tertiary structure is suggested by the fact that a single chaperone was able to amplify signals in different regions of the tmRNA molecule (chaperones A and D) and different chaperones amplified signals in the same region (chaperones A, D and E). Such an effect was also suggested by the generic increase of signal intensities of all non-overlapping probes, irrespective of location, in the presence of chaperones.

As expected, chaperones almost completely block the signals of the overlapping probes. Nevertheless, this effect is not absolute, as seen with chaperone E. The reason is still unclear. One possibility is weak chaperone binding, so the higher affinity capture probes outcompete it during hybridization. However, the chaperone has to have a sufficiently high affinity to outcompete the secondary structure of tmRNA successfully. This contradiction may indicate that at least in some cases, the factor limiting hybridization is not secondary but tertiary structure. Although intramolecular double-stranded regions are energetically weak, they fold the tmRNA molecule in such a way that some parts of it are not easily accessible. In such a case, the energetically much stronger target-probe hybridization is slow because the probability that a probe will hit its target area is very low, especially as the probes are immobilized on a surface. Chaperones were applied at a higher temperature at which there was no tertiary structure. Also, the relative concentration of chaperone molecules was much higher, both because they were applied in abundance and because they were free in solution. Thus, the chaperones could hybridize to any region, and if the temperature was lowered, the tertiary structures either did not form or were much weaker.

## Conclusion

We thus conclude that while the hybridization of tmRNA can sometimes be relatively slow at low temperatures, it can be significantly increased by using specific helper oligonucleotides (chaperones). The exact effect of certain helper nucleotides on the strength of the signal of certain capture probes depends on many factors, including probably the three-dimensional structure of the target molecule. The effect is not always local, meaning that a chaperone in the immediate proximity of the capture probe may not increase the signal strength, while one at a distant location might. As the structures of denatured nucleotide sequences cannot be precisely predicted at present, experimental verification of chaperones is necessary.

## Methods

### Bacterial strain and *ssrA*

The pCR^®^II-TOPO vector (Invitrogen, Carlsbad, CA, USA) containing *Streptococcus pneumoniae *ATCC 33400 tmRNA encoding *ssrA *was obtained from Dr. Barry Glynn, National University of Ireland, Galway, Ireland. The tmRNA gene was positioned into the vector under the transcriptional control of the T7 promoter sequence.

### *In vitro *RNA transcription

The *ssrA*-containing vector was linearized in 1× buffer R using HindIII restriction endonuclease (both reagents from Fermentas UAB, Vilnius, Lithuania). The reaction was carried out for 60 min at 37°C, followed by 15 min enzyme inhibition at 65°C. *S. pneumoniae *tmRNA was transcribed *in vitro *using 25 ng linearized vector and 20 U T7 RNA polymerase according to the manufacturer's recommendations. Briefly, final 1× reaction buffer contained 2 mM ATP, 2 mM CTP, 2 mM GTP and 1 mM UTP; 30 U RiboLock™ ribonuclease inhibitor was added to prevent possible RNA degradation. Aminoallyl-UTP (aaUTP) was added to 1 mM final concentration, making the final UTP:aaUTP ratio 1:1. A reaction volume of 25 μl was achieved by adding DEPC-treated water. All the reagents were purchased from Fermentas UAB. The transcription reaction was continued for 120 min at 37°C. *In vitro *synthesized RNA was purified using a Nucleotide^® ^RNA CleanUp Kit (Macherey-Nagel GmbH, Düren, Germany) according to the manufacturer's protocol. A final 60 μl of the material eluted was dehydrated in an RVC 2-25 CD rotational vacuum concentrator (Martin Christ GmbH, Osterode am Harz, Germany).

### Fluorescent labeling of RNA

Extra amine groups were incorporated into the tmRNAs during *in vitro *transcription by adding aaUTP. The amine-modified RNA was further labeled with the monoreactive fluorescent dye Cyanine™ 3-NHS (Cy3) (Enzo, Farmingdale, NY, USA). Cy3 (50 nmole) was diluted in 2 μl DMSO (Applichem, Darmstadt, Germany) and added to tmRNA diluted in 7 μl 0.1 M Na_2_CO_3 _(pH 9.0). The mixture of RNA and dye was incubated at room temperature for 60 min and the remaining excess Cy3 label was quenched by adding 3.5 μl 4 M H_2_NOH. After the coupling reaction, 35 μl 100 mM sodium acetate was added to neutralize the solution. The labeled RNA was purified with a NucleoSpin^® ^Kit and dehydrated in an RVC 2-25 CD concentrator.

### *Streptococcus pneumoniae*-specific microarray

Capture probes on the custom-made *S. pneumoniae-*specific microarray were designed using SLICSel 1.0 software http://bioinfo.ut.ee/slicsel. SLICSel is a program for designing specific oligonucleotide probes for detecting and identifying microbes. To ensure maximal probe specificity, SLICSel uses the nearest-neighbor thermodynamic model to calculate hybridization affinities for the intended target and non-target sequences. The microarray consisted of three *Streptococcus *family-specific and 94 *S. pneumoniae species-*specific probes covering almost the full length of the 335 nucleotide tmRNA molecule. Probe length varied between 9 and 26 nucleotides (average 16), melting temperature (Tm) between 53°C and 60°C (average 58°C) and binding energies (ΔG) with complementary tmRNA were predicted to be between -17 kcal/mol and -30 kcal/mol (average -23 kcal/mol) at 45°C and in 50 mM salt [Additional file 1]. In addition, 21 probes specific for other bacterial tmRNA sequences were designed to test the specificity of hybridization. The other bacteria included five further members of the *Streptococcus *family (Groups A, B, C, D and G), *Klebsiella pneumoniae *and *Moraxella catarrhalis*.

All the probes designed were tested using Mfold http://mfold.bioinfo.rpi.edu[29,30] to exclude those with potential secondary structures, and MegaBlast http://www.ncbi.nlm.nih.gov/blast/megablast.shtml[31] to eliminate possible cross-hybridization with unwanted targets, including tmRNAs from other species, bacterial DNA/RNA and human genomic DNA or RNA sequences. Three extra control probes with complementary fluorescent targets (spikes) were designed for normalization. Microarray probes with 5'amino modifications and C6 spacers were diluted in 100 mM Na_2_CO_3_/NaHCO_3 _(pH 9.0) to 50 μM final concentration and spotted on to SAL-1 Ultra microarray slides in Asper Biotech Ltd., Tartu, Estonia. Each slide contained four datapoints because two identical subgrids were spotted with duplicate spots.

### Chaperone design

**Figure 4:**
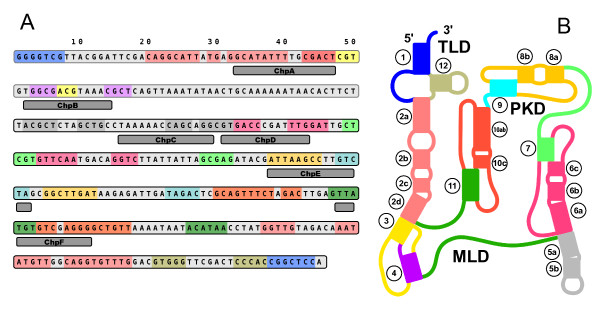
**tmRNA structure and chaperone positions**. **(A) ***S.pneumoniae *R6 tmRNA sequence [GenBank:NC_003098.1] with predicted helices highlighted in color and chaperone positions marked. Prediction and coloring according to tmRNA website [34]. **(B) ***E.coli *tmRNA secondary structure [35]. Highlight colors of helices are identical to panel A.

tmRNA molecules fold into complex structures of pseudoknots, tRNA-like regions and mRNA-like regions. A set of helper oligonucleotides ("chaperones") was designed with SLICSel to reduce the difficulty of hybridizing certain inaccessible regions of tmRNA. Six different chaperones were designed to bind to predicted secondary structure regions in *S. pneumoniae *tmRNA and prevent those intramolecular interactions (Figure 4). The complete set of chaperones is shown in Table 1. All the microarray probes, *spike-s *and chaperones used in the current work were ordered from Metabion, Mariensried, Germany.

**Table 1:** Helper oligonucleotides (chaperones) used in the current study and their characteristics.

	Position	Length	Sequence 5'-3'	GC%	Tm	ΔG
**ChpA**	33-47	15	AGTCGCAAAATATGC	40	53,8	-21,0

**ChpB**	52-64	13	GTTTACGTCGCCA	53,8	54,2	-20,0

**ChpC**	116-129	14	CCTGCTGGTTTTTA	42,9	56,9	-19,6

**ChpD**	131-143	13	CAAATCGGGTCAC	53,8	54	-20,1

**ChpE**	188-202	15	TAGACAAGGCTTAAT	33,3	54,6	-20,5

**ChpF**	248-261	14	CCCTCGACACATAA	50	62,5	-21,6

Tm and ΔG are calculated by program SLICSEL.

### Microarray experiment

One pmol of labeled RNA was resuspended in 80 μl microarray hybridization buffer (6× SSC; 0.5% SDS and 5× Denhardt's solution) together with *spike*-s (0.25 nM each). The hybridization mixture was heat-denaturated at 95°C for 5 min and snap-cooled on ice. The RNA hybridization melting curve was obtained by incubating for 4 h at temperatures ranging from 34°C to 70°C. Helper oligonucleotide experiments were conducted at 34°C with 10, 100 or 1000 pmol of chaperones added (making the ratios of tmRNA:chaperone ratios in solution = 1:10; 1:100; 1:1000); an equal amount of RNAse-free water (Macherey-Nagel) was added to the controls without chaperones. The effect of each helper oligonucleotide, individually and in a mixture of all six chaperones, was investigated. To determine the effect of hybridization time, RNA with chaperones was hybridized on to the microarray slides for 2-12 h. All hybridization experiments were conducted using an automated HS-400 hybridization station (Tecan Austria, Grödig, Austria). The complete hybridization protocol at 34°C is shown in Table 2. Prewash solution: 6× saline sodium citrate (SSC), 0.5% sodium dodecyl sulphate (SDS). Wash 1: 2× SSC. 0.03% SDS. Wash 2: 1× SSC. Wash 3: 0.2× SSC. All wash solutions and the prewash solution were warmed to 42°C. After hybridization, the slides were scanned using an Affymetrix 428 scanner (Affymetrix, Santa Clara, CA, USA), λ = 532 nm. Raw signal intensity data were analyzed using Genorama™ BaseCaller software (Asper Biotech). Microarray signals were rescaled to co-analyze data from different arrays by equating the average of the spike-specific signals from each microarray.

**Table 2:** Microarray hybridization protocol used in an automated HS-400 hybridization station.

		Temp. C°	Duration	Repetitions
**1**	Prewash	85	Wash: 60 s; Soak: 30 s	1

**2**	Probe injection	34		1

**3**	Hybridization	34	High agitation	1

**4**	1. wash	23	Wash: 90 s; Soak: 30 s	3

**5**	2. wash	23	Wash: 90 s; Soak: 30 s	3

**6**	3. wash	23	Wash: 90 s; Soak: 30 s	3

**7**	Slide drying	23	90 s	1

### Secondary structure prediction

An RNA/DNA folding package mFold was used to determine the tmRNA secondary structure. All degenerate nucleotides in the *S. pneumoniae *tmRNA sequence were substituted with N. All folding parameters were kept at default values. The ten most energetically advantageous structures were calculated for visual analysis of possible common motifs. The full-sequence energy diagram was also calculated. In addition, the probability of each nucleotide of tmRNA being in the hybridized state was calculated using the RNA analysis package sFold http://sfold.wadsworth.org[32,33] with default values.

## Competing interests

The authors declare that they have no competing interests.

## Authors' contributions

LK conceived and designed the study, analyzed and interpreted the results and wrote the manuscript. OS designed and was responsible for carrying out the microarray experiments and drafted the manuscript. SP participated in microarray experiments and helped to draft the manuscript. PP developed and tested probe selection algorithms and designed probes and chaperones. KT carried out the microarray experiments and performed Genorama analysis. AK conceived of the study, participated in its design and helped to draft the manuscript. MR provided financial and administrative support and participated in the design of the study.

All authors have read and approved the final manuscript.

## Supplementary Material

Additional file 1 **List of microarray probes**. List of all microarray probes (97 *S.pneumoniae *specific and 21 controls) used in hybridization. Click here for file
